# Marasmus

**Published:** 1894-08

**Authors:** Herman D. Marcus

**Affiliations:** Philadelphia, Late Resident Physician at the Philadelphia Hospital; 2263 North Twenty-first Street


### MARASMUS.

By HERMAN D. MARCUS, M. D., Philadelphia,
Late Resident Physician at the Philadelphia Hospital.

Marasmus, or simple atrophy, is essentially a disease of infancy, affecting children during their first years. The etiology of this disease is almost always due to injudicious feeding, or to a lack of cleanliness. In the group of injudicious feeding we may place both breast and bottle-fed children.

Breast-fed children may suffer from a lack of milk or from feeding on innutritious milk, while bottle-fed children almost always suffer from this disease when brought up on a poor quality of milk, the quantity being nearly always in excess. Breast-fed children are exposed to the natural changes in the mother's milk due to emotional disturbances, again, although the breast milk may be perfectly normal, the digestive organs of the infant may be in such a state as to demand a different nutrition, owing to the richness of the mother's milk.

In this age, we may truly say that bottle-fed children are gradually increasing in number, and we will, therefore, find a greater number of children suffering from malnutrition than in former years when a mother's pride rejoiced in giving her milk to her babe.

The essentials to a proper feeding of bottle-fed children are: (1) clean vessels ; (2) proper food.

Only too often we may find children fed on the very best preparations suffering from diarrhea, vomiting, and the like, when close investigations will show carelessly cleansed vessels. Thus the bottle will be refilled or the rubber nipple used over and over again without properly washing either, and on inspection we may find both bottle and nipple encrusted with curd and smelling abominably. The newer nipples, consisting of a long rubber and glass tubing, are undoubtedly the most wretched inventions against cleanliness. The caliber of these tubes is so narrow and the whole affair so complicated as to defy thorough cleansing. The plain rubber nipple and a small bottle without any corners inside are the most appropriate and the easiest cleaned.

The baby's food should, until the first year, be exclusively milk. No bread, meat, vegetable, or like food should be permitted, and the plea, "to get them used to everything," is a very poor one.

The digestive organs of an infant are not sufficiently developed to absorb such food, and the result of such a practice may soon be observed by the alimentary disturbances following.

In the first place of artificial foods must be put cow's milk. Cow's milk, when properly prepared, will be found the very best substitute for mother's milk. As cow's milk contains more caseine and less sugar than mother's milk, this should be equalized thus for new-born babies—the milk should be diluted equally with water to which sugar should be added. But despite all precautions as to proper amount of food, proper sterilization and regulation of intervals in feeding we may still find the infant ill-nourished.

In cases of marasmus in breast-fed children the cause will most probably lie in the poor quality of the milk. We first notice a gradual emaciation. The face becomes pinched and anxious-looking, the lips anemic; the child becomes fretful; during nursing the child grasps the nipple with fervor and will suck the milk ravenously. In such cases no special organic defects can be noticed.

Marasmus in bottle-fed children is accentuated by the rapid emaciation, the peculiar wasted face—appearing like that of an old person. The appetite is ravenous and cannot be satisfied. The extremities, in fact the whole body, emaciate rapidly until the child has the appearance of a skeleton.

Although constipation is most frequent, diarrhea accompanied by vomiting may set in. At times nervous symptoms may be observed, but this is rare, most infants lying as if in a stupor. The temperature is nearly always subnormal.

In the treatment of this affection the diet, of course, is the most important factor. In early cases regulation of the diet is all that is required; more advanced cases may demand medicinal treatment. It is of the greatest importance to give in such cases easily assimilable and highly nutritious food. Probably the best of the so-called food preparations are Carnrick's lactopreparata and soluble food. The former closely resembles human milk and should be given to infants up to seven or eight months, while the latter consists of lactopreparata and dextrinated wheat, and should be given to older infants. These foods are highly nutritious, very easily digested and will be found of the greatest help in the treatment of marasmus.

Inunctions of oil and internally bicarbonate of soda and bismuth in such cases which are complicated by diarrhea and vomiting, or small doses of calomel, followed by castor oil or some aperient, if constipation is present, will be found of the greatest benefit. Cod liver oil, preferably the pure oil, may be given as soon as the stomach is able to retain it.

Fresh air and extreme cleanliness are hygienic precautions which all infants need, especially those afflicted with marasmus.

2263 North Twenty-first Street.

---

In Toronto a health officer requested the dismissal from school of a child suffering with phthisis, and this exercise of authority precipitated a legal contest. The judge sustained the action of the official, claiming the disease to be contagious.—*Maryland Med. Journal.*

